# The Use of 360-Degree Video in Developing Emotional Coping Skills (Reduced Anxiety and Increased Confidence) in Mental Health Nursing Students: A Protocol Paper

**DOI:** 10.3390/nursrep12030052

**Published:** 2022-07-17

**Authors:** Caroline Laker, Pamela Knight-Davidson, David Hawkes, Paul Driver, Maxine Nightingale, Ann Winter, Andrew McVicar

**Affiliations:** Department of Health, Education, Medicine and Social Care, School of Nursing, Chelmsford Campus, Anglia Ruskin University, Chelmsford CM1 1SQ, UK; pamela.knight-davidson@aru.ac.uk (P.K.-D.); dave.hawkes@aru.ac.uk (D.H.); paul.driver@aru.ac.uk (P.D.); maxine.nightingale@aru.ac.uk (M.N.); winter230@btinternet.com (A.W.); andy.mcvicar@aru.ac.uk (A.M.)

**Keywords:** 360-degree video, nurse education, stress and coping, mental health nurses, emotional distress, cognitive re-appraisal, solution-focused brief therapy, VERA

## Abstract

Higher education institutions are uniquely placed to introduce emotional coping skills to promote resilience in pre-registration nurses in order to reduce anxiety and increase confidence before they enter clinical placement for the first time. In this qualitative study, we will explore the use of a 360-degree video in developing skills for coping. The participants will be mental health nursing students. We will develop a 360-degree video in collaboration with a mental health service user. All participants will watch the video. A sub-group will receive a supportive clinical supervision discussion within a cognitive reappraisal/solution-focused/VERA framework. We will record the experiences of the participant to explore: (1) how students felt about the use of 360-degree video, as an education tool to build skills of resilience; (2) whether the students involved felt more confident and less anxious about the situation in the video as a result of participating in the cognitive reappraisal/solution-focused/VERA supervision discussion.

## 1. Introduction

In U.K. healthcare settings, there are a number of factors that might contribute to nurses’ experiences of stress at work such as excessive workload, shortages of time, staff and resources [1,2], ineffective leadership, low levels of control in the workplace and conflict (which may be between colleagues or directed by patients/clients towards staff) [3,4].

In mental health settings, there may be additional psychological and emotional stressors for nurses which are distinct from other health settings, by their nature and degree [5]. Within these (often) locked environments, where service users are not necessarily confined to beds through illness, a social milieu emerges, which is founded on interactions between the nursing staff and the service users [6]. Working in these fluid and frequently chaotic settings can have negative consequences, as mental health ward staff express higher levels of emotional exhaustion than staff in other settings, as well as interaction anxiety around patient contact [5,7,8]. Interaction anxiety may be linked to a need for self-preservation, as staff attempt to protect themselves from emotional distress, either subconsciously or knowingly [3,5,7,9], which may detract from the supportive, caring behaviours required in care delivery.

It is clear that in mental health nursing, more research is needed to help staff develop resilient dispositional, psychological and social attributes, which are fundamental in forming effective therapeutic working relationships with patients [5]. We also recognise this need in pre-registration nurse education especially prior to entering a practice placement for the first time. Given ‘feeling in control’ is a corner stone in coping with stress [10], empowering student nurses to explore their emotions in stressful working situations might help students to develop emotional coping skills and build resilience.

**The 360-degree video as an educational tool:** This qualitative study protocol will outline an approach to explore the combined use of simulation-based and experiential learning approaches to support student nurses to learn about how to handle and respond to emotional distress. The beneficial value of simulation in dealing with psychosocial challenge and potential confrontation is established [11], but it is difficult to achieve in a safe and realistic environment particularly when working with inexperienced students. The 360-degree video provides an innovative and safe opportunity as a simulation-based educational tool to help students manage their stress and anxieties when placed in a challenging situation. Using experiential learning to acquire cognitive behavioural therapy skills has already proven successful amongst student nurses [12]. In this study, the clinical supervision strategy will allow students a supported and experiential opportunity to talk through and reflect on how they might respond to a service user in distress. The 360-degree video is described in Appendix A). 

**Developing emotional coping skills: techniques to reduce anxiety and build confidence in student nurses:** There are a number of studies which show a more positive adjustment to stress if an individual is able to reappraise their emotional responses [13,14,15], which can also positively influence psychological health [16,17,18].

In this study, the clinical supervision strategy will focus on reducing anxiety using cognitive reappraisal, an evidence based therapeutic principle which draws from cognitive behavioural therapy (CBT) [19] and building confidence using solution-focused therapeutic techniques [20,21,22,23,24,25].

Most cognitive behavioural approaches aim to build skills that individuals can apply to new situations [19]. Cognitive reappraisal aims to decrease the experience of negative emotions and has become an integral part of many cognitive behavioural approaches to reduce anxiety and change negative thought and behaviour cycles [19,26]. Cognitive reappraisal is a structured process, focused on the antecedents of an emotional response, which allows an individual to identify the thoughts and beliefs that are affecting how they feel [27]. Thereafter, the meaning of a situation is reformulated in order to reduce its emotional impact [28]. Change is effected by introducing new learning experiences that help people challenge their initial negative automatic thoughts about a situation and rehearse new ways of coping and acting [29].

Students will also be taught a communication strategy based on the validate, emotion, reassure, action (VERA) cycle for communication; an existing model of training and supervision to promote confidence [30,31] which draws from solution-focused brief therapy (SFBT) principles [20,21,22,23,24,25]. VERA has been used as a structured cycle of communication, clinically with Alzheimer’s disease and dementia, in Accident and Emergency settings to de-escalate aggression and in work with psychosis and voice hearing. In a study evaluating VERA, which was implemented on two older adult in-patient wards, staff expressed more confidence to engage with service users and felt that the approach broadened their repertoire of therapeutic interventions [31].

The VERA cycle for communication has four key stages [31]:

(1) To provide validation for behaviour or communication and to avoid negative and unhelpful assumptions that may create barriers to engagement;
(2) To engage with the emotional content of what is communicated;
(3) To offer reassurance and to promote a sense of safety;
(4) To take the communication forwards into meaningful activity or to reflect on the interaction.

**Preparation work:** an immersive video scenario will be developed using 360-degree technology. The film will be approximately 5 min in length and will be set in an in-patient unit/ward. Actors will play the role of service users and nurses on the ward. The main focus will be on a mental health service user exhibiting emotional distress and the viewer will have the opportunity to follow this character around inside the ward.

Permission to film in a vacant ward setting will be sought from a hospital setting in a UK healthcare Trust.

Involvement will be sought from an individual who has lived experience of the mental health services during the creation of the video to ensure that the final product is sensitive, appropriate, well rounded and upholds ethical standards [32].

## 2. Methods

**Study Aim:** The overall aim of this study is to help prepare mental health students for potentially stressful experiences in practice. Participants will experience an immersive 360-degree video showing a service user experiencing emotional distress within a mental health setting and a subgroup will be supported by a tutor to explore, appraise and reappraise their feelings and emotional responses, within a cognitive reappraisal/solution-focused/VERA framework.

**Objectives:**

1. To evaluate the use of 360-degree video as an educative tool.
2. To evaluate whether using a clinical supervision discussion builds confidence and reduces anxiety in relation to stressful clinical interactions, using a combined cognitive reappraisal/solution-focused/VERA framework.

**Figure 1:**
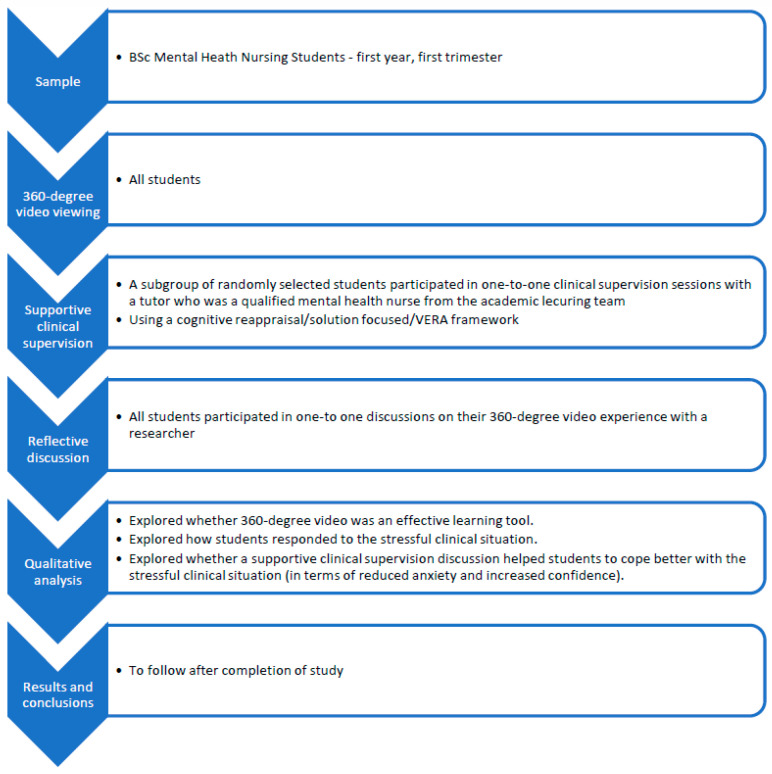
Study outline.

**Procedure (see Figure 1):**

**Sample:** Two cohorts of first year student nurses, enrolled on a BSc Mental Health Nursing course and yet to experience a practice placement, will be invited to participate. The intention is to recruit around N = 30 participants. Demographic data will be collected from the sample (e.g., age, gender, and ethnicity). Whether participants had prior exposure to mental health settings will also be assessed.

## 3. Exploring 360-Degree Video as an Educational Tool—All Participants

To meet objective 1, participants will follow a service user showing signs of emotional distress in a ward setting, using an immersive 360-degree video to create a real-life experience. Participants will be video-recorded to provide both visual and audio data.

These data will allow an exploration of whether 360-degree video is an effective learning tool. All participants will view the video, and answer questions on the 360-degree experience, which will provide an opportunity to reflect on the technology and the immersive experience.

## 4. Exploring A Supportive Clinical Supervision Using a Cognitive Reappraisal/Solution-Focused/Vera Framework—With a Subgroup of Participants

To meet objective 2, a subgroup of around half the recruited participants will be randomly selected to receive the supportive clinical supervision, within a cognitive reappraisal/solution-focused/VERA framework. Participants will be supported to consider what skills they already have to help them produce effective responses to the distressed service user. This group of students will watch the video, then participate in the supportive clinical supervision discussion, and then watch the video again.

The procedure for the supportive clinical supervision will be as follows:

After viewing the film for the first time, the supervisor will ask participants to:

Identify emotions: as part of the process of cognitive reappraisal, the participants will describe how they felt whilst watching the scenario. They will rate the intensity of their emotional responses using a scale from 0 (not intense at all) to 10 (the most intense I have ever felt).

After the film, the supervisor will support the students to evaluate their thoughts. At this stage, prompts will include:

- What is going through my mind as I am feeling this emotion?
- What am I telling myself about this situation?
- What am I afraid might happen?

Next, participants will be asked to rate how strongly they hold these beliefs on a scale of 1–10.

Then participants will be asked to rate their levels of confidence and anxiety. Scaling questions will be asked (e.g.,):

- How anxious do you feel about this situation on a scale of 0–10?
- What would move your anxiety levels down the scale?
- How confident are you that you would be able to deliver an intervention that would help the service user move on from this situation on a scale of 0–10?
- What would move your confidence levels further up the scale?

Participants in the subgroup will also be supported to explore how they might respond in the situation using solution-focused brief therapy skills guided by the supervisor based on the VERA framework [30,31]. The supervisor will support the participants to formulate appropriate responses to questions such as:

- Why might the service user in the video be feeling this way?
- What do you think they are trying to communicate?
- What response might you give to this distressed service user?
- What clinical decisions might you undertake based on the scenario?

The cognitive reappraisal and solution-focused evaluation will be recorded by the supervisor using a worksheet [33,34] (see Appendix B).

## 5. Qualitative Data Analyses

Social interaction is a key element of mental health nursing and, given this study concerned an interaction between pre-registration mental health students and a distressed client in an acute setting, this study was underpinned by social phenomenological theory [35]. Qualitatively, this research will explore:

1. Whether pre-registration mental health nursing students consider 360-degree video to be an effective learning tool.
2. How pre-registration nursing students interpret and respond to stressful clinical situations.
3. Whether working through a process of cognitive appraisal and clinical supervision (to introduce concepts of SFBT and the VERA framework) helped students to cope better with a stressful interaction (in terms of reduced anxiety and increased confidence).

This study is also concerned with using video to capture pseudo real-world data. The audio-visual data allows comparisons between visible conduct as well as verbal content to inform our understanding of how student nurses respond in complex, stressful social interactions which include a distressed client. Given the data will provide an opportunity to explore body language, actions, movements and spoken words, all will be transcribed and analysed using a thematic content analysis which has the flexibility to cross data formats [36]. Greater attention will be paid to the ‘themes’ that develop through the coding process than in a standard content analysis, to allow a deeper understanding of the interactional phenomena [37]. Nvivo 11 software will be used for the analyses.

## 6. How the Research Will Be Communicated to the Wider Community/Research Outputs

The findings of this study will be written up for publication.

**Outputs:** An interactive digital overlay, containing additional didactic information, questions and multimedia, will be added to the 360-degree video. This will be developed into an online educational tool for nursing students. This tool will be fully compatible with Canvas, a course management system that supports online learning and teaching.

## 7. Ethical Considerations

This research involves human participants, that is, student nurses enrolled on the BSc Mental Health Nursing course. We will not require a gatekeeper for this research as the students are known to the department. There will be no financial incentives to participate in this research; however, students will benefit educationally from the experience of participation. Ethical approval has been awarded for this study (reference: NNM-SREP-18-012). Approvals were sought through the University ethics application system.

We are mindful that participation in this research could reveal a health/mental health issue. As we are interested in how stress might be remediated to improve students’ clinical experiences in practice, this will not be considered an incidental finding. Details of internal and external support agencies will be provided to the participants.

Service users will facilitate the creation of the 360-degree video. A service user researcher experienced in facilitating service user involvement in research will provide support for those involved.

## 8. Conclusions

In this study, the 360-degree scenario will be focused on a mental health service user in a state of emotional crisis, to enable student nurses in the field of mental health to explore what skills they have to enable them to cope and offer support. However, if successful, this approach might be tailored to other fields of nursing to allow the exploration of stressors in different settings. It may also beneficial in providing insight into how to work with mental health crisis for all fields of nursing.

## Appendix A

**Synopsis of 360-degree video:** The video depicts a woman exhibiting signs of mental distress in a mental health ward. The entire video is 5 min and 45 s in length, with a 60 s period of no activity at the start to allow the viewer time to adjust to the equipment. There are two scenes. Scene 1: The woman is shown coming through a door and up to the camera, to which she speaks. She expresses a wish to engage and states that she is upset about being kept on the ward and wishes to leave. Her level of distress increases, and she walks to the doors at the end of the corridor and tries to leave but finds that the doors are locked. She comes back to the camera and throws a cup at it before going into her bedroom where she sits down on the floor and cries. It is clear from her behaviour that she is in a state of despair. Scene 2: The woman wishes to enter a lounge area which is labelled as a ‘female only area’. She is surprised to find two men inside this room talking to each other. She asks them why they are there, and they are not receptive. She expresses concern that they are not leaving and shows her frustration with the staff who do not appear to be helpful.

## Appendix B

Reappraisal Task Worksheet
Thank you for participating in this task. You will be shown a 360-degree experiential video. The scenario depicts an interaction between you, the viewer, and a service user called Mary. She will interact with you and others on the ward.

1. Scenario

How would you describe the difficult or stressful experience that you have just viewed? One sentence is sufficient.

2. Feelings

Describe the feelings/emotions that you felt during the experience
A. Limit your description to the a few words or phrases that best describe the feelings.
B. Record the intensity of the feelings. Use a scale from 0 (no feelings) to 10 (very intense feelings).

- How intense were your feelings during the experience?_________
- How intense are your feelings about the experience now?_______
- How anxious did you feel when viewing this situation?__________

What would have moved your anxiety levels down the scale?

3. Thoughts

A. As you were viewing the scenario, what was going through your mind?

Consider the following:

- How did I look at this experience?
- What did it mean to me?
- What did it mean about me/my imminent future?

B. As you were feeling the emotion, (stated above), what was going through your mind?
Consider the following:

- What were you telling yourself about the situation?
- What were you afraid might happen?

SCALING
Taking your thoughts and feelings into consideration, how reasonable does your view seem? Use a scale from 0 (not reasonable) to 10 (very reasonable)_______________________________________________
Now that you have considered your view, how reasonable does it seem? (0–10)______________________________________________________
How confident are you that you could deliver an intervention that would help the service user move forwards on a scale of 0–10_______________________________________________________
What would move your confidence levels further up the scale?

4. Reframing feelings and thoughts using a Solution Focused approach

Think about a situation in your past where you dealt successfully with an anxiety provoking incident.

- What did you do then to successfully manage the situation?
- What did you say, keep in mind, feel or remember that helped you solve the situation in a positive way?

What qualities have you shown in life and work that suggest that you can cope well in this sort of situation?
Think about an individual that you have helped successfully in the past. If they were here, what would they say to you about your skills/abilities to deal with this scenario?
Knowing your strengths and your life and work experiences so far, what would you do / say in this situation if it happened again?
How would you help Mary to feel listened to and show her you value her as a whole person?

- What is the emotional content of what Mary is saying in this situation?
- What do you think she is feeling?

If you had to reassure Mary and give her some sort of positive compliment what would that be?
How could you and Mary work together to address her concerns and to keep the conversation going?
NOW RE-WATCH THE VIDEO
RE-SCALING
How do you feel about the experience now after considering these points-of-view?
If you consider your thoughts and feelings about the scenario now, how reasonable were your initial views? Use a scale from 0 (not reasonable) to 10 (very reasonable).__________________________
How anxious do you now feel about this situation on a scale of 0–10?______________________________________________________
How confident are you now that you would be able to deliver an intervention that would help Mary move on from this situation on a scale of 0–10?___________________________________________________
**Qualitative questions about the 360-degree experience**
With these questions, we are not testing your knowledge. We are only interested in your opinion. There is no right answer. It is not a test/nor does it form any part of your assessment…….

1. In the video, the service user is talking to someone. In your view, who was that person?
2. How did you relate with/to the interaction?
3. How able were you to relate to the service user?
4. How did you feel overall about the 360-degree video experience?
5. What emotions were evoked for you, when watching the video?
6. Can you describe how you felt whilst watching the video?
7. How realistic did you think the video was?
8. Can you elaborate on your relationship with what was happening in the scenario?
